# CASE REPORT Journey of a Noma Face

**Published:** 2010-06-30

**Authors:** Colin Yi-Loong Woon, Karen Wei-Ee Sng, Bien-Keem Tan, Seng-Teik Lee

**Affiliations:** Department of Plastic, Reconstructive and Aesthetic Surgery, Singapore General Hospital, Outram Road, Singapore 169608

## Abstract

**Objective:** Noma, or cancrum oris, is rare in developed countries. Surgeons are likely to encounter this disease only in the context of a medical mission. While it is tempting to approach noma sequelae as an oncologic resection, an understanding of the disease process will reveal that the challenge is quite different. In addition, unlike the oncologic patient who desires rapid return to an aesthetically normal facies, the adult noma patient with chronic history of noma sequelae may be more accepting of a functional but less aesthetic outcome. **Methods:** We describe a noma patient with soft-tissue losses involving right cheek, nasal ala, upper lip and oral commissure, and severe trismus who underwent staged reconstructive surgery. **Results:** The objectives of temporomandibular joint release, facial defect coverage, correction of occlusal cant, and restoration of lower facial symmetry were met. The final planned stage of reconstruction was declined as the patient had regained sufficient self-confidence to participate in social activities. **Conclusions:** Surgeons from developed countries rarely encounter adult patients with noma sequelae. While reconstructive principles remain the same, noma reconstruction must be approached differently from oncologic resection and a staged approach is often necessary. Although complete correction may be planned to restore function and aesthetics, the noma patient may eventually be satisfied with a functional but less aesthetic outcome.

Surgeons in developed countries rarely encounter noma except in the context of a medical mission. Noma, from the Greek word *nome*, means “to devour”.^1^^-^4 Adult patients with noma sequelae display gross destruction of facial tissue. The disease is found in less developed regions of Africa, Asia, and South America.^5^,^6^ In addition to having little access to modern medicine, these patients often battle the triad of malnutrition,^2^,^7^,^8^ poor oral hygiene, and periodontal disease.^2^,^4^ Mortality, once as high as 70% to 90% (ref 2) has decreased to 10% (ref 5) with modern antibiotics and improved nutrition, leaving more children surviving into adulthood with large defects.^9^

Although a reconstructive surgeon may be keen to apply the principles of oncologic reconstruction to noma surgery, it must be noted that the 2 are dissimilar and a slightly different approach is necessary.

We present a case of an adult patient with noma sequelae managed by a staged approach. Although complete correction was initially planned, in accordance with the patient's requests, the last stage was abandoned and a functional but less aesthetic result accepted. In the discussion, the importance of preoperative planning, staged surgery, a multidisciplinary approach, and close postoperative follow-up is emphasized.

## CASE REPORT

**Figure 1:**
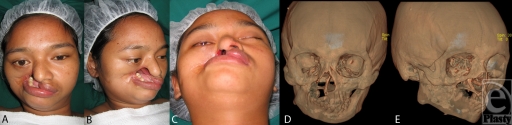
(*a*). Marked facial asymmetry, pronounced occlusal cant and right orbital dystopia. (*b*) and (*c*) Soft-tissue loss involving right cheek and oral commissure, right upper lip and ala, exposing the vomer and nasal septum. (*d*) and (*e*) Computed tomographic images showing right temporomandibular joint ankylosis, right zygomaticomandibular fusion, right ascending ramus hypoplasia and coronoid process hypertrophy (from temporalis contracture),^5^ loss of the right maxilla and maxillary teeth, vomer and one-third of hard palate.

A 20-year-old Laotian woman presented with a large facial defect and bilateral trismus (Fig 1). At 4 years of age, she came down with a high fever. A foul-smelling, fulminating ulcer developed over her right cheek and evolved into a black eschar that ulcerated. She gradually developed temporomandibular ankylosis (“constriction des mâchoires”)^10^^-^12 with interlocked left-sided teeth and was unable to open her mouth to feed or talk. She fed by inserting food through the facial defect and manually grinding it against her remaining right mandibular teeth.

Examination and imaging revealed marked facial asymmetry, pronounced occlusal cant, and right orbital dystopia (Fig 1a). Soft-tissue losses involved the right cheek and oral commissure, right upper lip and ala, exposing the vomer and nasal septum, leaving a 4.5 cm by 3 cm defect (Figs 1b and 1c). Bony abnormality included right intra- and extraarticular ankylosis (temporomandibular joint ankylosis and zygomaticomandibular fusion, respectively), loss of the right maxilla and maxillary teeth, vomer and one-third of hard palate, right ascending mandibular ramus hypoplasia, and a hypertrophic coronoid process from temporalis muscle contracture (Figs 1d and 1e).

Because of the financial limitations of the sponsoring organization, it was intended that the initial more complicated stages of surgery were performed at our institution, with later stages to be performed in her home country.

**Figure 2:**
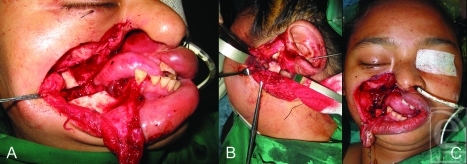
(*a*) First stage scar excision and zygomaticomandibular release with horizontal mandibular ramus osteotomy. Maximal incisal opening (MIO) of 0.5 cm was achieved. (*b*) and (*c*) Left condylectomy, coronoidectomy and fascia lata interpositional arthroplasty (Fig 2b) to attain final MIO of 2.5 cm. Bilateral release resulted in a larger facial defect (Fig 2c).

At the first operation, the cheek scar was excised and the right temporomandibular joint and zygomaticomandibular fusion released with a horizontal ramus osteotomy, achieving maximal incisal opening (MIO) of 0.5 cm (Fig 2a). The left side was explored (Fig 2b), revealing extraarticular (fibrous) trismus, flattening of the left mandibular condyle, glenoid fossa, and articular eminence. Left condylectomy, coronoidectomy, and fascia lata interposition arthroplasty (Fig 2b) was performed. Complete bilateral release resulted in a larger facial defect measuring 7 cm by 5 cm (Fig 2c). An intraoral orthodontic appliance was fashioned as a bite block to maintain the MIO postoperatively.

The soft-tissue defect was resurfaced with an ipsilateral free anterolateral thigh (ALT) flap with skin paddle dimensions measuring 8 cm by 6 cm. She was on the bite block continuously for 1 week, followed by nocturnal bite block for 6 months, and then intensive jaw physical therapy was instituted.

**Figure 3:**
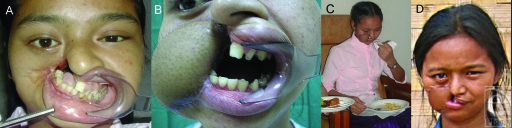
(*a*) and (*b*) Pre- and postoperative occlusal photographs showing maximal incisal opening increase from 0 cm to 2.5 cm. (*c*) Eating with utensils. (*d*) Appearance at 1 year postoperatively.

Second-stage reconstruction at 2 months involved limited debulking, repositioning of the ALT flap and refashioning of the right oral commissure. Compared with preoperative occlusal photographs (Fig 3a), she had improved MIO of 2.5 cm (Fig 3b), allowing her to eat with utensils (Fig 3c). She returned home 1 month later.

At 3-month and 1-year mission visits (Fig 3d), we found that she had regained enough self-confidence to interact with peers and participate in village activities. She no longer hid behind a scarf when eating.

The original plan was to include a third stage, to be performed in her home country. This was to involve reverse Estlander flap reconstruction of the right upper lip and right oral commissure, ALT flap debulking, right nasal ala reconstruction, and rehabilitation with a maxillary prosthesis. However, she expressed satisfaction with the existing results and declined further intervention.

## DISCUSSION

This patient displays a Montandon type IV facial defect^3^,^9^ that is typical of noma sequelae. Type IV defects, the largest and most common noma lesion, presents with loss of whole cheek, lips, maxilla, palate, and malar bone.^9^ These defects are usually unilateral, although up to 15% of patients may have defects that cross the midline.^13^ Although previously uncommon, the incidence of type IV defects is rising owing to increased survival as a result of initiation of antibiotics early in the course of childhood disease.^9^

Surgery is targeted at restoring normal speech, oral competence, and acceptable cosmesis.

The surgery differs from reconstruction of defects following ablative oncologic resection in the following ways. (1) In the adult noma patient, compound tissue losses (skin, soft tissue, bone) are commonplace. (2) Remnant tissue is left scarred and distorted with loss of normal anatomical reference points. Because noma occurs in early childhood, the bony defect and soft-tissue scar affect facial skeleton and dental development, producing gross asymmetry.^9^,^14^ (3) Nutritional deficiencies are commonplace.^2^,^7^^-^9,14 Preoperative optimization must include nutritional assessment and correction of these deficiencies. (4) Complications, including poor healing and infection, are common in the immediate postoperative period. Hence, a lengthy observation period is necessary prior to discharge and return to the country of origin.

## PRINCIPLES OF MANAGEMENT

We adhered to the following pillars of management. (1) Nutritional deficiencies must be addressed before surgery is considered. This includes rehydration, correction of electrolyte abnormalities, and dietary supplements with iron, folic acid, vitamin C and B complex as necessary.^14^ (2) Surgery should be performed after a period of disease quiescence of at least 6 to 18 months.^4^,^13^,^14^ A longer period is preferred to allow for correction of nutritional deficiencies, for demarcation of involved tissue, and for better determination of the role of reconstruction. As noma is a childhood disease, surgery can usually be delayed and emergency surgery is only necessary for control of intractable hemorrhage.^14^ (3) Both soft tissue and bony losses must be addressed and the chronological sequence in which this is undertaken is important. First, ankylosis must be released and the mandible mobilized before resurfacing the facial defect.^1^,^4^,^13^ The chronological sequence is crucial because the resultant facial defect is invariably larger after release, and premature defect closure may deprive them of their sole means of nutrition.^1^ (4) Postoperative rehabilitation to prevent recurrence of ankylosis is as important as the surgery itself.

## TRISMUS

In our patient, the most deleterious sequelae of noma present was bilateral trismus^14^,^15^ which impairs nutrition, oral hygiene, and speech. Trismus was attributed more to false (extraarticular) ankylosis than true (intraarticular) ankylosis,^15^ as evidenced from computed tomographic (CT) scan findings. Contributing factors include an anterior scar band, loss of the oral lining, and scarring of masticatory muscles and joint capsule,^1^,^9^,^15^ with bony zygomaticomaxillary fusion on the right. Fusion arises from ossification of a dense block of submucous scar tissue uniting the ramus of the mandible to the maxilla or zygomatic arch.^14^

Following bilateral release, a few measures may help prevent recurrence: (1) complete bilateral release; (2) flap interposition between raw bone surfaces on one side, and interpositional arthroplasty on the other^10^;(3) postoperative immobilization with bite blocks or external fixator for controlled elongation of the mandible elevator muscles^11^; and (4) intensive supervised rehabilitation.^11^ Although her final MIO of 2.5 cm is far from ideal, it was functional and allowed social feeding. The optimal final MIO of 4 cm was unattainable owing to loss of pterygoid muscle function to disuse atrophy and fibrosis. In spite of these preventive measures, recurrence (from partial to complete reankylosis) is likely^9^,^15^ and continued follow-up with local physicians is advised, although not always possible.

## SOFT TISSUE

Soft-tissue resurfacing aims to restore both inner and outer linings of the cheek.^11^ For outer coverage, various locoregional, pedicled, and free cutaneous and myocutaneous flaps^3^,^9^,^16^ have been described, including forehead,^4^ platysma,^1^,^9^ deltopectoral,^9^,^17^ pedicled and free latissimus dorsi,^4^,^9^ free rectus, radial forearm,^9^,^18^ serratus anterior, and parascapular flaps.^4^,^19^ We chose the workhorse ALT flap for its size, long vascular pedicle, tissue characteristics, and because it could be harvested by 2 teams working simultaneously.^20^ The ALT flap leaves minimal morbidity and is easily concealed under clothing, important in young females of marriageable age.^15^ Intraoral defects may be addressed with split-skin grafts, or left to mucosalize,^11^ as in this case. Some authors, however, are opposed to the idea of split-skin grafts and secondary epidermalization because of secondary contraction contributing to trismus recurrence,^9^,^14^ preferring instead local rotation flaps.

For such cases with complex defects, complete correction should be staged, with aesthetic refinements performed during subsequent operations,^9^ although some authors advocate a single-stage approach.^16^ The initial, most difficult stage is best performed in a location where a multidisciplinary team^9^ (plastic/craniofacial surgeon, anesthesiologist, orthodontist, physical therapist, nursing), modern facilities (radiological imaging, operating microscopes, surgical intensive care units), and equipment for management of a difficult airway (eg, fibreoptic intubation, tracheostomy capability) are readily available.^9^ It is also important that the patient only returns home when all wounds have healed and perioperative complications have been treated.

Subsequent, less complex stages can be performed in the patient's home country, recognizing that certain resources (eg, microsurgical facilities) may be limited or unavailable.

## CONCLUSION

Noma sequelae comprise bony and soft-tissue losses that affect both aesthetics and function. Complex defects should be treated in a staged fashion with the most complex initial stages performed in better-equipped institutions and less complex stages done on home ground. Although complete correction may be planned to restore function and aesthetics, the noma patient may eventually be satisfied with a functional but less aesthetic outcome.
